# Modulating reward and aversion: Insights into addiction from the paraventricular nucleus

**DOI:** 10.1111/cns.70046

**Published:** 2024-09-18

**Authors:** Shihao Huang, Cuijie Shi, Dan Tao, Chang Yang, Yixiao Luo

**Affiliations:** ^1^ Hunan Province People's Hospital The First‐Affiliated Hospital of Hunan Normal University Changsha China; ^2^ National Institute on Drug Dependence and Beijing Key Laboratory of Drug Dependence Research Peking University Beijing China; ^3^ Department of Neurobiology, School of Basic Medical Sciences Peking University Health Science Center Beijing China; ^4^ College of Forensic Medicine Hebei Medical University Shijiazhuang China; ^5^ School of Medicine Hunan Normal University Changsha China; ^6^ Key Laboratory for Birth Defects Research and Prevention of the National Health Commission Hunan Provincial Maternal and Child Health Care Hospital Changsha China

**Keywords:** addiction, AVP, CRF, OXT, paraventricular nucleus

## Abstract

**Background:**

Drug addiction, characterized by compulsive drug use and high relapse rates, arises from complex interactions between reward and aversion systems in the brain. The paraventricular nucleus (PVN), located in the anterior hypothalamus, serves as a neuroendocrine center and is a key component of the hypothalamic–pituitary–adrenal axis.

**Objective:**

This review aimed to explore how the PVN impacts reward and aversion in drug addiction through stress responses and emotional regulation and to evaluate the potential of PVN as a therapeutic target for drug addiction.

**Methods:**

We review the current literature, focusing on three main neuron types in the PVN—corticotropin‐releasing factor, oxytocin, and arginine vasopressin neurons—as well as other related neurons, to understand their roles in modulating addiction.

**Results:**

Existing studies highlight the PVN as a key mediator in addiction, playing a dual role in reward and aversion systems. These findings are crucial for understanding addiction mechanisms and developing targeted therapies.

**Conclusion:**

The role of PVN in stress response and emotional regulation suggests its potential as a therapeutic target in drug addiction, offering new insights for addiction treatment.

## Graphical abstract

The paraventricular nucleus regulates drug addiction directly by modulating reward and aversion systems or indirectly by affecting stress response and emotional regulation. In this process, the corticotropin‐releasing factor, oxytocin, arginine vasopressin, rostral ventrolateral medulla, neuropeptide Y, agouti‐related peptide, and pro‐opiomelanocortin neurons are involved in the modulation of drug addiction.

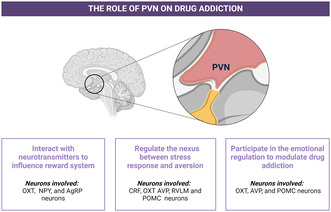

## 1 INTRODUCTION

Drug addiction is a complex brain disease characterized by compulsive drug‐seeking behaviors and use despite severe adverse consequences.^1^, ^2^, ^3^ The rewarding effects of addictive drugs initially drive addiction by targeting the mesolimbic dopamine (DA) system, leading to increased extracellular DA concentrations in the nucleus accumbens (NAc) and eliciting pleasure.^4^ This mesolimbic DA system is considered the “common pathway” for drug addiction, crucial for the development of addictive behaviors.^5^ Over time, drug use results in adaptive changes in synaptic plasticity within key brain regions, including the prefrontal cortex (PFC), amygdala (AMY), hippocampus, thalamus, and hypothalamus.^6^, ^7^, ^8^ These changes enhance or attenuate synaptic transmission, leading to the consolidation of drug‐associated memories and compulsive drug use.

Addictive drugs not only produce rewarding effects but also trigger stress and aversive responses due to their negative effects. This phenomenon is attributed to the shared neural circuits and neurotransmitters between the brain's reward and aversion pathways, such as the extended AMY and the mesolimbic system.^9^ Negative effects induced by addictive drugs are involved in various stages of addiction, potentially exacerbating adverse symptoms through negative reinforcement, leading to increased drug use. For example, during the initial stages of drug use, addictive substances can activate corticotropin‐releasing factor (CRF) neurons in the ventral tegmental area (VTA) and NAc, leading to stress and aversive responses.^10^, ^11^, ^12^, ^13^ As drug use continues, CRF neurons in the VTA and PFC contribute to the transition to compulsive drug use. In later stages of addiction, CRF neurons in the extended AMY and dorsal striatum mediate negative emotional states and drug‐seeking behaviors.^13^ Repeated exposure to drugs can also lead to tolerance to the drug's aversive effects and increase susceptibility to subsequent use of the same or other addictive substances.^14^, ^15^, ^16^ For instance, adolescent rats pre‐exposed to tetrahydrocannabinol showed increased heroin self‐administration in adulthood and were more prone to relapse triggered by drug cues or drug priming, suggesting that pre‐exposure to an addictive drug can increase susceptibility and craving for other substances.^17^

The interplay between reward and aversion mechanisms underlies the persistent and relapsing nature of addiction. Addictive drugs produce intense rewarding effects and negative reinforcement effects, leading to compulsive drug use, withdrawal symptoms, and psychological cravings. These negative effects can impair the brain's reward system and enhance the anti‐reward system, altering susceptibility to addiction, sustained drug use, and stress‐induced relapse.^15^, ^18^ The reward mechanism is often linked to DA release, while the aversion mechanism may involve changes in neurotransmitters such as cortisol (CORT), forming the biological basis of addictive behaviors. Given the inadequacies of existing addiction treatments that predominantly target the brain's reward and anti‐reward systems, there is a strong rationale for investigating novel brain regions implicated in the pathophysiology of addiction.

The paraventricular nucleus (PVN), a critical neuroendocrine center located in the anterior part of the hypothalamus adjacent to the sides of the third ventricle, is crucial in regulating stress responses, energy balance, and emotions.^19^, ^20^, ^21^ It produces neuropeptides and neurohormones such as CRF, oxytocin (OXT), and arginine vasopressin (AVP), modulates the hypothalamic–pituitary–adrenal (HPA) axis, and interacts with other brain regions to influence systemic physiological states.^22^, ^23^, ^24^ The capacity of PVN to modulate the stress axis through CRF production and govern social behaviors and emotional states via OXT and AVP underscores its intricate involvement in the dynamics of reward and aversion.^15^, ^16^, ^25^, ^26^ In recent years, the PVN has gained increasing attention in addiction research due to its crucial role in the pathophysiology of the reward and aversion systems.

The PVN is a critical component of the HPA axis and is instrumental in regulating stress responses.^27^, ^28^ In the context of addiction, the regulation of the HPA axis by PVN is particularly significant. Stress is a key factor that influences the development, maintenance, and relapse of addictive behaviors.^29^ Chronic drug use can dysregulate the HPA axis, leading to heightened stress sensitivity and hormonal imbalances, which can exacerbate addiction.^15^, ^30^ This dysregulation often results in an increased propensity for relapse in recovering addicts, particularly when exposed to stressors or drug‐related cues.^31^ The interactions of PVN with DA pathways also indirectly influence the brain's reward system, and repeated drug exposure can alter the function of PVN, disrupting these pathways and affecting the reward system's response to drugs and other stimuli.^5^, ^32^, ^33^ This disruption may contribute to the compulsive drug‐seeking behaviors characteristic of addiction. Additionally, the role of PVN in emotional regulation through hormones like OXT and AVP indicates its involvement in the negative reinforcement effects of drug addiction. Alterations in the production and release of these hormones can affect social bonding and stress‐coping mechanisms, which are crucial in the development and maintenance of addictive behaviors.^34^, ^35^ For instance, changes in OXT levels have been associated with social behaviors and stress responses, which are often dysregulated in addiction.^25^, ^26^ The PVN's involvement in stress regulation makes it a vital area of study for understanding the “dark side” of addiction, which encompasses the negative emotional states associated with withdrawal and craving. Moreover, the PVN contains various neuron types that interact with the reward and aversion circuits in the brain. These interactions can directly affect addiction by modulating reward and aversion or indirectly influence addiction by regulating stress and emotional states. This positions the PVN as a critical hub in the neurobiological network of addiction, offering new avenues for research and potential therapeutic targets. By reviewing these aspects, we aim to underscore the importance of the PVN in addiction research and its potential to provide deeper insights into the mechanisms driving addictive behaviors.

In summary, the involvement of PVN in both reward and aversion systems underscores its potential significance in the treatment of drug addiction. By modulating the stress response and influencing emotional regulation, the PVN could be a target for novel addiction therapies. This article explores the relationship between the PVN and addiction, focusing on the roles of different neuronal types within the PVN in mediating reward and aversion effects and the implications for addiction treatment.

## 2 THE PVN REGULATES BOTH REWARD AND AVERSION VIA VARIOUS FACTORS TO MODULATE DRUG ADDICTION

Stress response and emotion regulation interact with the reward and aversion systems of addiction through multiple factors.^29^, ^36^ Specifically, the stress response affects the DA system through the HPA axis and CRH, altering sensitivity to reward and aversion, and thereby impacting addictive behaviors.^31^ Emotional regulation is indirectly related to the risk of addiction, with effective emotional regulation mitigating the negative reinforcement mechanisms that drive addiction.^36^ The PVN, as a key regulator of the stress response, modulates the HPA axis and interacts with other brain regions, significantly affecting the development of addiction.^37^ Therefore, understanding these complex interactions is crucial for developing more effective addiction treatments and stress management strategies.

### 2.1 PVN interacts with neurotransmitters and key reward‐related areas to influence the reward system

The rewarding effect of addictive drugs is the initial driving force behind drug addiction. Addictive drugs initially target the mesolimbic DA system during the onset of drug use, leading to a rewarding effect by increasing extracellular DA concentrations in the NAc.^4^ The mesolimbic DA system is currently regarded as playing a crucial role in the development of addictive behaviors.^5^ Additionally, addictive drugs also activate other related brain regions, such as the PFC, AMY, hippocampus, thalamus, and hypothalamus,^7^, ^8^ leading to abnormal alteration in synaptic plasticity among these brain areas. This involves either enhancement or attenuation of synaptic transmission, resulting in adaptive changes in the targeted brain regions.^6^ PVN not directly a part of the traditional reward circuitry, significantly influences the reward system through its interactions with key reward‐related areas and neurotransmitters.^37^, ^38^ The regulation of stress hormones in PVN, particularly its impact on the HPA axis, indirectly affects DA pathways, which are central to the reward system.^37^, ^39^

Recent studies have demonstrated that the PVN also plays a role in the reward mechanism, primarily mediated by OXT neurons.^40^ It has been found that OXT can bidirectionally regulate an individual's approach and avoidance behaviors toward peers and strangers in social contexts, underscoring its critical role in the recognition and interpretation of social information.^41^, ^42^ Cho et al. observed in a monogamous prairie vole model that OXT is closely associated with the formation of pair bonds.^43^ OXT facilitates the formation of intimate relationships among animals and reduces defensive behaviors in social contexts, thereby promoting greater proximity and social interaction.^44^ Additionally, OXT can reverse stress‐induced social avoidance, leading stressed rats to exhibit more social approach behaviors.^45^ The OXT gene knockout mice exhibit social cognitive deficits, which can be reversed by microinjection of OXT into the AMY region.^46^ Huang et al. discovered that blocking OXT receptors in the VTA reduced mice's interest in social interaction without affecting cocaine reward memory formation. Activating PVN OXT neurons increased social behaviors toward pups but did not influence other activities like cage preference or lever pressing for food. Manipulating axon terminals of PVN OXT neurons projecting to VTA DA neurons altered social inclination. Electrophysiological studies showed that OXT receptor agonists enhanced VTA DA neuron firing, indicating the PVN OXT‐VTA circuit promotes social behaviors by increasing DA release in the NAc during social encounters.^40^ Moreover, the increase in OXT release in non‐social contexts does not affect cocaine‐induced motivational and reinforcing behaviors. These findings underscore the complex interplay between OXT signaling and social behaviors and the specificity of the effects of OXT on social versus non‐social contexts, providing valuable insights into the neurobiological mechanisms underlying social interactions and addiction. Additionally, stress‐induced activation of the PVN can lead to altered DA release in areas like the NAc, thereby influencing reward‐related behaviors and responses, and the connections of PVN with other brain regions involved in reward processing, such as the VTA and PFC, further underscoring, its role in modulating the complex interplay between stress, reward, and addiction.^37^, ^40^, ^47^, ^48^ This intricate relationship highlights the significance of PVN in stress response and shaping how rewards are perceived and sought after, particularly under varying emotional and stress conditions.

### 2.2 PVN regulates the nexus between stress response and aversion to modulate drug addiction

Addiction is not solely driven by the reward system; it is also closely related to the aversive responses.^49^ Aversive plays an important role in the addiction process through withdrawal symptoms and negative reinforcement mechanisms.^50^ On the one hand, when addicts do not have access to addictive substances, they experience intense stress responses and physical discomfort known as withdrawal symptoms, which are mediated by the AMY and CRF system.^9^ These negative effects exacerbate addiction through negative reinforcement, to reduce stress or avoid negative emotional states.^51^ For instance, withdrawal‐induced stress responses are mediated by the CRF system in the AMY and PFC, contributing to compulsive drug use and relapse.^9^, ^13^ During stress, activity in the AMY and PFC increases, making individuals more sensitive to negative stimuli.^30^, ^52^, ^53^ This enhanced aversion response prompts the addict to continue using the addictive substance to escape the discomfort, thereby forming a negative reinforcement mechanism. Research has elucidated that acute stress markedly upregulates the expression of immediate early genes, such as c‐Fos and phosphorylated extracellular regulated protein kinases 1/2 (ERK 1/2) in the PVN, indicating the critical role of PVN in mediating the stress response.^54^, ^55^ This response includes the activation of the HPA axis via the PVN, which facilitates the release of adrenocorticotropic hormone (ACTH) and subsequently elevates serum corticosteroid levels.^39^ In addition, studies have shown that CRH neurons in Central AMY (CeA) regulate negative emotions during withdrawal through their projection to VTA, suggesting that stress may play a role in addiction.^56^ Consequently, PVN regulates the nexus between stress response and aversion to modulate drug addiction.

The involvement of PVN in stress response to modulate drug addiction, particularly through the CRF neuronal pathway, has garnered significant scientific interest.^37^ Within this pathway, neuroendocrine neurons project to the pituitary gland, releasing CRF and thus modulating the stress response via the HPA axis.^57^, ^58^ Kim et al. have discovered that CRF neurons in the PVN can be directly activated by a variety of aversive events, while their activity significantly decreases in response to rewarding stimuli, indicating that PVN CRF neurons (PVN ^CRF^) are responsive to both reward and aversion.^59^ It has been found that nearly all addictive drugs can directly or indirectly activate PVN CRF neurons, thereby triggering the activation of the HPA axis.^60^ This activation may constitute one of the mechanisms through which addictive drugs induce negative emotional states and lead to drug tolerance following repeated use.^61^ Furthermore, the CRF system is involved in the entire process from the initiation of addiction to compulsive drug use, with CRF in different brain regions participating in this process.^13^ PVN CRF neurons are particularly involved in the initial stages of drug use; acute cocaine exposure can enhance the activity of PVN CRF neurons and activate the HPA axis,^62^ while chronic cocaine administration can lead to a reduction in CRF expression within the PVN.^63^ This suggests that chronic cocaine administration may inhibit the function of PVN CRF neurons, potentially serving as one of the mechanisms by which the body develops tolerance to the negative effects of addictive substances. Moreover, the PVN orchestrates the stress response through various neuropeptide signaling pathways, including the reciprocal regulation of stress by OXT and AVP. OXT is known for its anxiolytic and antidepressant properties, whereas AVP can elicit anxiety and depressive behaviors.^64^ The equilibrium between these neuropeptides is crucial for maintaining emotional homeostasis.^65^ OXT also influences stress response by modulating CRF. Research indicates that stressors such as social defeat or forced swimming trigger OXT release, which can amplify CRF‐induced corticosteroid secretion and inhibit CRF gene transcription, thereby attenuating the stress response.^66^, ^67^ Furthermore, OXT neurons can establish synaptic connections with CRF neurons, enabling the regulation of diverse behavioral responses through intracerebral neural microcircuits.^68^ Additionally, the PVN receives regulatory inputs from other brain regions, such as neuropeptide S (NPS) neurons from the locus coeruleus (LC), which project to PVN OXT neurons (PVN^OXT^).^69^ NPS stimulates these neurons to release OXT, and its anxiolytic effect is contingent upon the presence of OXT within the PVN. The blockade of this effect by an OXT receptor antagonist underscores the significance of the PVN OXT system in mediating NPS‐induced anxiety relief.^69^ These findings underscore the complexity of the PVN's role in stress response, highlighting its capacity to integrate diverse neuronal signals and modulate the body's adaptation to stress through a multifaceted network of interactions.

### 2.3 PVN participates in emotional regulation associated with the response to reward and aversion to modulate drug addiction

Emotional regulation is closely related to stress response, with the PVN playing a key role in regulating this response.^64^, ^70^ The PVN controls the release of stress hormones such as cortisol by activating the HPA axis.^37^ Poor emotional regulation leads to excessive and persistent stress responses, increasing the risk of depression and anxiety.^71^ Research shows a two‐way relationship between depression and addiction.^72^ Inadequate emotional regulation is a significant factor in addiction formation, withdrawal, and relapse.^73^ The use of addictive substances to cope with depression and anxiety contributes to addiction. During withdrawal, addicts often experience severe emotional swings, including depression and anxiety, which are important triggers for relapse.^74^ After withdrawal, the stress response heightens negative emotions, prompting a return to addictive substances or behaviors. The PVN plays a key role in regulating these negative emotions, influencing individual emotional regulation and relapse by modulating stress responses.^75^, ^76^

Specifically, the PVN is crucial in emotional regulation through its influence on hormones like oxytocin and vasopressin.^77^ These hormones are integral to social bonding, stress response, and emotional stability. Oxytocin often called the “love hormone,” enhances feelings of trust, empathy, and social connection, significantly affecting emotional states and responses to reward and aversion.^78^, ^79^ Vasopressin, while also involved in social behaviors, is more closely linked to aggression and territorial behaviors.^80^, ^81^, ^82^ The balance and interaction of these hormones, regulated by the PVN, are essential for maintaining emotional homeostasis. Furthermore, the connections of PVN with other brain regions involved in emotional processing, such as the AMY and PFC, enable it to integrate hormonal signals with neural information, influencing emotional responses and mood states.^22^, ^83^ This integration is vital for understanding how emotional contexts affect decision‐making, stress responses, and behaviors related to addiction and reward. The role of PVN in emotional regulation is pivotal in both normal psychological functioning and psychiatric disorders, where emotional and social behaviors dysregulation is prominent.

The above background suggests that PVN may play a therapeutic role in drug addiction by modulating reward or aversion effects. The following sections will primarily provide a comprehensive review of the roles of different types of PVN neurons in reward and aversion.

## 3 THE NEURAL MECHANISM OF PVN MODULATES DRUG ADDICTION

The PVN, together with the rest of the midline and intralaminar thalamic nuclei, is primarily composed of excitatory neurons that facilitate communication between various cortical and subcortical regions.^84^, ^85^, ^86^ Neuronal diversity within the PVN is notable, with three main types of neurons: magnocellular, parvocellular, and long‐projecting neurons. The parvocellular subset contains a variety of neuron types, including AVP, OXT, rostral ventrolateral medulla (RVLM), and DA. In contrast, the magnocellular neurons primarily produce AVP, OXT, and nitric oxide (NO). Surrounding the parvocellular subset are interneurons that modulate their activity. These interneurons primarily release γ‐aminobutyric acid (GABA), glutamate, and NO, forming numerous synaptic connections with presympathetic neurons in the PVN.^87^, ^88^

**FIGURE 1:**
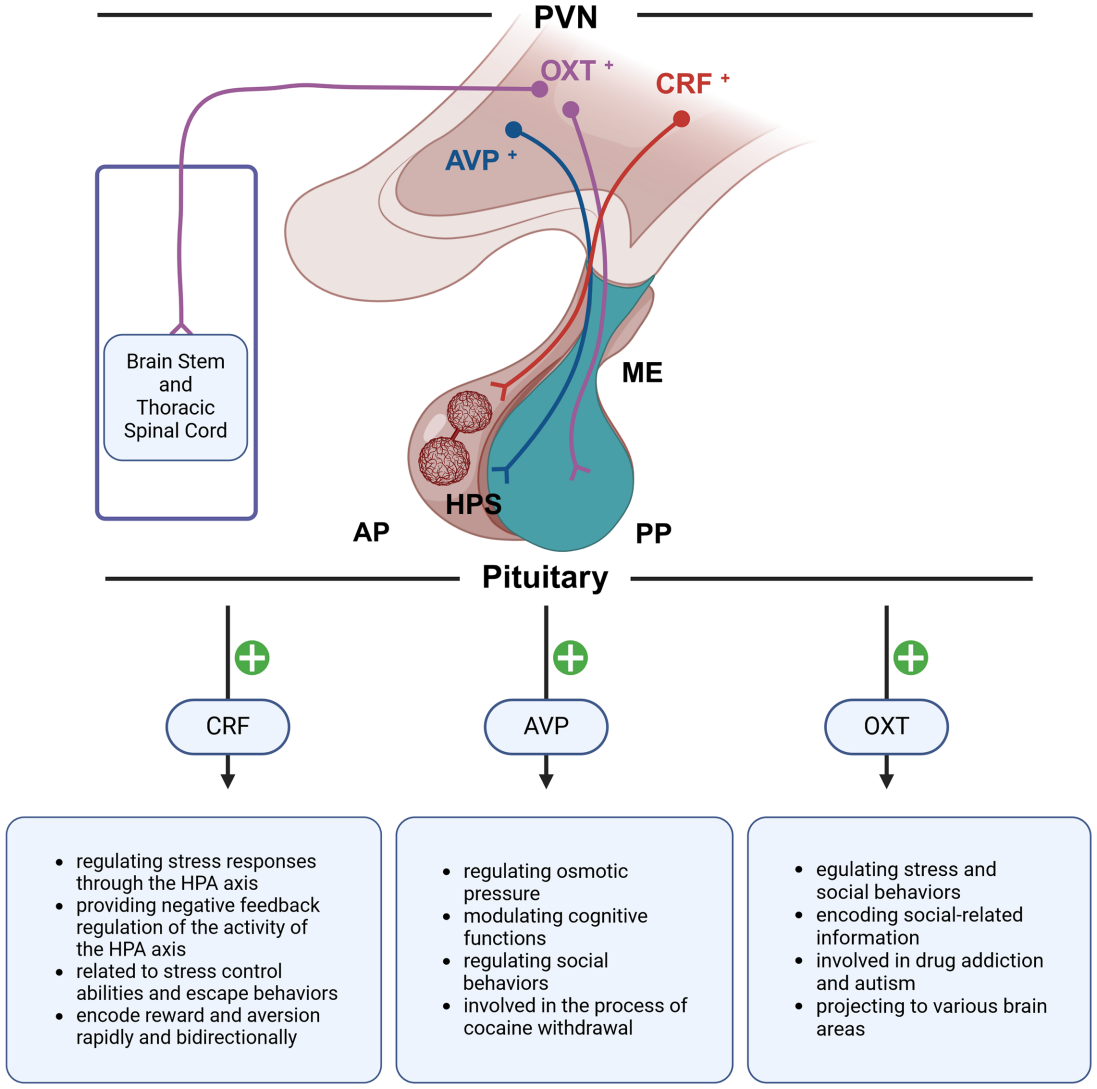
The role of various neuronal types in the PVN. The diagram reveals the role of different neuronal types in the PVN in both reward and aversion, including corticotropin‐releasing factor (CRF), oxytocin (OXT), and arginine vasopressin (AVP) neurons. AP, anterior pituitary; AVP+, arginine vasopressin neurons; CRF+, corticotropin‐releasing factor neurons; HPS, hypophyseal portal system; ME, median eminence; OXT+, oxytocin neurons; PP, posterior pituitary; PVN, paraventricular nucleus.

Specifically, we focus on CRF neurons, OXT neurons, AVP neurons, and other neurons that may play roles in the addiction process (Figure 1). CRF neurons primarily influence addiction by regulating the stress response.^37^ OXT neurons are more involved in emotional and social behaviors, affecting the motivational aspects of addiction.^89^ AVP neurons are related to water−salt balance and blood pressure regulation, influencing the physiological responses in addiction.^90^ By focusing on different neuron types, we can comprehensively understand the complex mechanisms of PVN in addiction and better guide intervention and treatment strategies for addiction. The roles of CRF, OXT, AVP, and other related neurons of PVN in stress response, mood regulation, reward processing, and aversive response are elaborated below, revealing the complex influence of PVN activity on the multifaceted dynamics of addiction.

### 3.1 The role of corticotropin‐releasing factor (CRF)‐expressing neurons in PVN in regulating reward and aversion

CRF is a 41‐amino acid CRF that is secreted into the pituitary portal system through the hypothalamic–pituitary‐neurosecretory system. The neurons in the PVN that secrete CRF can be categorized based on cell morphology into small and large cell types. The CRF neurons with neuroendocrine functions in the PVN are of the small cell type. These neurons project their axonal terminals to the median eminence to secrete CRF, which regulates the release of ACTH and β‐endorphin from the pituitary gland.^58^, ^91^ It is widely accepted that this signaling pathway serves as the “gatekeeper” of the HPA axis, playing a crucial role both under basal and stress conditions.^21^ Additionally, small cell CRF neurons in the PVN can also secrete other substances such as AVP, cholecystokinin, enkephalin, vasoactive intestinal peptide/peptide histidine isoleucine, neuropeptide Y, and angiotensin.^92^ Research has also found that some large cell AVP and OXT neurons in the PVN can secrete CRF. However, these neurons play a minimal role in regulating the release of ACTH. Instead, they may regulate the secretion of AVP and OXT through autocrine or paracrine mechanisms involving CRF. CRF receptors are divided into type 1 (CRFR1) and type 2 (CRFR2) receptors, each playing different roles.^21^, ^93^

Extensive research has been conducted on the regulation of stress responses by CRF neurons through the HPA axis.^58^, ^94^ The activity of these neurons is modulated not only by neurotransmitters such as OXT, AVP, and CRF but also by inputs from other brain regions. CRF neurons receive both GABAergic and glutamatergic synaptic transmissions, and the ratio of excitatory/inhibitory inputs plays a significant role in regulating these neurons. Miklós et al. have found that chronic stress can remodel the synaptic inputs to CRF neurons in the rat PVN.^95^ Rats experiencing chronic stress showed a significant increase in the total number of synaptic terminals on PVN CRF neurons, but the overall excitatory/inhibitory ratio remained unchanged. Instead, there was an increase in the number of GABAergic synapses connecting to the dendrites of CRF neurons, reducing their connections to the soma.^95^, ^96^ Simultaneously, there was an increase in excitatory synapses connecting to the soma, leading to enhanced CRF release and sustained activation of the HPA axis.^95^ Therefore, PVN CRF neurons can exhibit long‐term synaptic plasticity changes during chronic stress, suggesting their potential involvement in the pathogenesis of neuropsychiatric disorders such as anxiety and depression.

Furthermore, Ramot et al. discovered that PVN CRF neurons also released CRF within the PVN, acting on CRFR1 neurons through local microcircuits to provide negative feedback regulation of both their activity and that of the HPA axis, which first confirmed the presence of a class of neurons expressing CRFR1 in the mouse PVN.^97^ After chronic stress in mice, glucocorticoids (GCs) can increase the expression of CRFR1 in the PVN through a positive feedback mechanism. The high expression of PVN CRFR1 was accompanied by a significant increase in CORT levels, suggesting its involvement in chronic stress by regulating the HPA axis. Subsequently, the team further explored how PVN CRFR1 neurons affect the HPA axis. They found that PVN CRFR1 neurons are closely adjacent to PVN CRF neurons and can form synaptic connections with them, regulating the activity of CRF on the HPA axis through local microcircuits. Under chronic stress, CRF released by CRF neurons can activate CRFR1 neurons, which then inhibit the activity of CRF neurons through GABAergic synapses. Selective knockout of PVN CRFR1 neurons can enhance the activity of CRF neurons and increase corticosterone release during stress, while also slowing the reduction of corticosterone levels when stress ceases.^98^ Therefore, Justice and his team revealed a negative feedback regulation mechanism of local microcircuits in PVN CRF neurons during stress.

All the aforementioned studies have focused on the role of PVN CRF neurons in regulating the body's stress response through the neuroendocrine action on the HPA axis pathway. Recent research has also discovered that these neurons have certain hormone‐independent functions in stress‐related behaviors, such as grooming behaviors in mice.^99^ It has been found that PVN CRF neurons can also be involved in the active responses of animals to stress. For instance, mice facing danger can activate PVN CRF neurons while emitting warning signals to alert their peers. The activation of these neurons can also initiate rapid defensive behaviors, suggesting that PVN CRF neurons might be related to stress control abilities and escape behaviors.^59^, ^100^, ^101^, ^102^ Daviu et al. used optogenetics and fiber photometry to show that PVN CRF neurons regulate mice's responses to predator threats. Inhibiting these neurons shifts behaviors from escape to freezing during threat signals. PVN CRF neuron activity spikes during escape but returns to normal when the area is safe. Mice trained to control stress showed increased PVN CRF neuron activity during threats, while those without control showed a delayed response, indicating their role in anticipation of danger and adaptability to stress.^28^ This might be one of the reasons why individuals exhibit negative coping or learned helplessness in subsequent challenges after experiencing severe trauma. Apart from stress, PVN CRF neurons are also involved in the body's response to reward and aversion. Research by Kim et al. found that all aversive cues could activate PVN CRF neurons, while rewarding stimuli could rapidly inhibit their activity. Furthermore, optogenetic activation or inhibition of PVN CRF neurons could not only induce conditioned place aversion or preference in mice but also counteract the conditioned place preference (CPP) or aversion produced by natural stimuli.^59^ These results all indicate that PVN neurons have the rapid and bidirectional capability to encode reward and aversion.

### 3.2 The role of oxytocin (OXT) neurons in PVN in regulating stress and social behaviors

Oxytocin (OXT) is a neurohypophysial hormone composed of a structure of nine amino acids and is highly conserved throughout evolution.^40^ It is primarily synthesized in the PVN and the supraoptic nucleus (SCN) and secreted by the posterior pituitary. OXT is widely expressed across different species and plays important roles in various behaviors, such as sexual arousal,^103^ maternal behaviors,^104^ mating,^105^ as well as social cognition and social memory.^106^ Anxiety, stress, and social stimuli can all induce the synthesis and release of OXT in the brain.^107^ Physiological stimuli can also trigger the secretion and release of OXT through different pathways. For instance, sexual activity, maternal behaviors, and social preference can lead to increased secretion of OXT in the central nervous system, while labor, lactation, and orgasm can elevate the levels of OXT in the peripheral nervous system.^108^ Recent research has also found that OXT plays a significant role in neuropsychiatric disorders, such as addiction and autism.^89^, ^109^

PVN OXT neurons, which are large cell‐type neurons in the PVN, primarily function in regulating stress and social behaviors.^110^, ^111^ This regulation may be achieved through the local release of OXT within the PVN, modulating the excitability of PVN CRF neurons. Jamieson et al. found that OXT and CRF neurons are highly intermingled within the PVN, suggesting the possibility of local intercellular communication. Further, using in vitro electrophysiological recordings, they discovered that OXT administration could reduce the frequency of spontaneous excitatory postsynaptic currents (sEPSCs) in CRF neurons and had a slight and delayed inhibitory effect on spontaneous inhibitory postsynaptic currents (sIPSCs). These results indicate that OXT can inhibit the excitability of CRF neurons through synaptic transmission, which might be a potential mechanism for OXT's role in stress regulation.^112^

PVN OXT neurons play a crucial role in encoding social‐related information.^113^, ^114^, ^115^ Resendez et al. demonstrated that chemogenetic activation of these neurons significantly enhanced social exploration behaviors in mice during social choice tests, while inhibition of these neurons eliminated social preference. Using two‐photon calcium imaging, it was also found that social stimuli significantly activated PVN OXT neurons, and they exhibited different patterns when encoding social and non‐social stimuli. This suggests that they mediate social information in environmental stimuli. In the autism animal model, Shank3b knockout mice showed a reduction in the number of PVN OXT neurons. Administration of an OXT receptor agonist improved social impairments, indicating that PVN OXT neurons play an important role in adaptive social behaviors.^116^

Additionally, PVN OXT neurons are significant in drug addiction. Leong et al. found that OXT administration significantly reduced lever‐pressing behaviors induced by drug cues in cocaine‐addicted rats. Immunohistochemistry results showed a significant increase in the co‐labeling rate of Fos + and OXT neurons in the PVN following OXT administration.^117^ Kohtz et al. observed a significant reduction in the activity of rat PVN OXT neurons on the first day of cocaine withdrawal and the first day of extinction. OXT administration also reduced lever‐pressing behavior on the first day of extinction and after extinction when induced by cues.^89^ These results suggest that the aversive effects produced by cocaine can inhibit the activity of PVN OXT neurons, and enhancing their activity can eliminate cocaine‐seeking behaviors.

PVN OXT neurons can exert different effects by projecting to various brain areas. For instance, the PVN OXT to the prelimbic cortex (PrL) circuit is involved in anxiety and social behaviors. He et al. found that paternal deprivation before weaning increased anxiety‐like behaviors and reduced social preference in adult prairie voles, accompanied by significant inhibition of PVN OXT neurons and the PrL, as well as a marked reduction in OXT receptors in the PrL.^118^ Injecting OXT into the PrL could eliminate anxiety‐like and social resistance behaviors. Further, optogenetic activation of the PVN OXT‐PrL circuit (PVN^OXT^‐PrL) produced similar effects, while optogenetic inhibition of this circuit induced anxiety‐like and social resistance behaviors in normal voles. These results suggest that paternal deprivation before weaning can cause damage to the PVN OXT‐PrL circuit (PVN^OXT^‐PrL) in prairie voles, leading to emotional and social disorders. Restoring the function of this circuit through pharmacological or optogenetic means can eliminate anxiety and antisocial behaviors, indicating that the PVN OXT‐PrL circuit (PVN^OXT^‐PrL) plays a significant role in emotional and social disorders.

### 3.3 The role of arginine vasopressin (AVP) neurons in PVN in participating in the modulation of reward and aversion

AVP, also known as antidiuretic hormone, is a cyclic structure 9‐peptide hormone secreted by neurons in the PVN and the SCN.^119^ It travels via the hypothalamic–pituitary tract to be released into the systemic circulation from the posterior pituitary. The primary function of AVP is to regulate the homeostasis of plasma osmolality, thereby maintaining fluid balance in the body. It also regulates renal water excretion and thirst according to the body's needs. The role of AVP in maintaining the homeostasis of body plasma osmolality is irreplaceable by other hormones, and its deficiency can lead to diabetes insipidus.^120^, ^121^ There are three types of AVP receptors: V1aR, V2R, and V1bR (also known as V3R). Among these, the AVP receptors in the central nervous system are primarily V1a and V1b types.^122^ Recent research has found that AVP can be involved in the modulation of reward and aversion to a certain extent.

Since the 1970s and 1980s, there has been debate about whether AVP can directly affect the central nervous system. Subsequent research found that in addition to regulating osmotic pressure, AVP also has roles in modulating cognitive functions. De Wied et al. investigated the effects of AVP and found that central administration of Arg8 and its related peptides, such as pyroglutamate (pGlu), and cytochrome (Cyt) AVP (4–8), could influence animals' passive avoidance behaviors. This was the first evidence that AVP could directly act on the central nervous system to alter animal behavior.^123^

Following this, Winslow and others studied prairie voles and discovered that the function of AVP in the brains of these animals was involved in maternal behaviors. Additionally, AVP was found to regulate selective aggression and partner preference behaviors, indicating that AVP plays a key role in the monogamous mating behaviors of prairie voles.^124^ Clinical studies have also found that intranasal administration of AVP can improve social impairments in children with autism. Parker and colleagues conducted a randomized, double‐blind, placebo‐controlled parallel study with 30 children with autism. They evaluated the efficacy and tolerance of a 4‐week intranasal AVP treatment. The study found that intranasal administration of AVP, compared to a placebo, significantly enhanced the social abilities of children with autism. It also alleviated their anxiety symptoms and repetitive behaviors. Furthermore, AVP administration was well‐tolerated and produced almost no side effects.^125^ These results suggest that AVP has promising potential in regulating social behaviors. Social behaviors are closely related to emotional regulation and, in turn, influence addictive behaviors.^126^, ^127^ Therefore, by examining the role of AVP in social behaviors, we can indirectly understand its potential mechanisms in addiction.

Currently, there is limited research on the involvement of PVN ^AVP^ in addiction. Zhou et al. found that AVP in the PVN is also involved in the process of cocaine withdrawal. After rats underwent self‐administration training and then acute (1 day) to chronic (14 days) cocaine withdrawal, the expression levels of AVP mRNA in the PVN were found to continuously increase. The use of opioid receptor antagonists increased AVP mRNA in the PVN of the control group rats but did not affect rats undergoing cocaine withdrawal. This suggests that the inhibitory effect of opioids on PVN AVP neurons (PVN ^AVP^) is reduced after cocaine withdrawal.^90^ These results indicate that PVN AVP neurons may play a certain role in drug addiction.

### 3.4 The role of other neurons in PVN in participating in the modulation of reward and aversion

#### 3.4.1 RVLM neurons

The RVLM is a critical brainstem region involved in the regulation of cardiovascular functions.^128^, ^129^ Recent studies have also implicated RVLM neurons in the modulation of stress‐induced hypertension.^130^ The RVLM neurons project to various brain regions, including the PVN, influencing the hypothalamic–pituitary–adrenal (HPA) axis and autonomic responses.

Zhang et al. find a significant increase in M2 muscarinic receptors within RVLM during the peak period of withdrawal.^131^ Their findings suggest that heightened RVLM activity can trigger stress responses that are physical dependence. Furthermore, the excitation of putative adrenergic (C1) cells of RVLM during opiated withdrawal contributes to autonomic activation.^132^ These results indicate that PVLM is a potential target for therapeutic intervention in opiate‐induced withdrawal treatment.

#### 3.4.2 NPY/AgRP neurons

Neuropeptide Y (NPY) and agouti‐related peptide (AgRP) neurons in the arcuate nucleus of the hypothalamus (ARC) are known for their role in regulating feeding behaviors and energy homeostasis.^133^, ^134^ These neurons project to the PVN and are involved in the modulation of reward‐related behaviors.^135^, ^136^

Studies have shown that NPY can reduce drug‐seeking behaviors and withdrawal symptoms. Specifically, changes in PVN‐NPY neurotransmission may play a functional role in the food intake and weight‐modulating effects of nicotine.^137^ Furthermore, AgRP, which antagonizes melanocortin receptors, has been shown to influence the reward circuitry, thereby affecting addiction‐related behaviors. Olszewski et al. found that the primary effect of AgRP of PVN is to cause an increase in food intake to satisfy energy needs.^138^ Besides, Taylor et al. further elucidated that NPY and AgRP increase consummatory feeding responses, highlighting their role in the reward mechanisms related to feeding and potential addiction.^139^

#### 3.4.3 POMC neurons

Pro‐opiomelanocortin (POMC) neurons in the ARC play a crucial role in the regulation of energy balance, stress response, and reward processing. POMC neurons release α‐melanocyte‐stimulating hormone (α‐MSH), which binds to melanocortin receptors and modulates various physiological processes, including addiction.^140^

Biglari et al. identified functionally distinct POMC‐expressing neuron subpopulations in the hypothalamus, revealing the complex role these neurons play in various behaviors, including those related to addiction.^141^ Specifically, POMC is activated by nicotine and related to anxiety symptoms of alcohol‐dependent patients.^142^, ^143^ Moreover, epigenetic changes in the POMC promoter are associated with alcohol and tobacco dependence.^144^, ^145^ It is interesting to investigate whether such an effect depends on the PVN region. Furthermore, De Solis et al. demonstrated the reciprocal activity of AgRP and POMC neurons in coordinating the control of feeding and metabolism, which may also extend to mechanisms of addiction and reward.^146^

## 4 CONCLUSION

The comprehensive review of the PVN's role in reward and aversion systems underscores its critical involvement in drug addiction. PVN is involved in regulating different processes of addiction at various levels, both functionally and with the neuronal subtypes. The regulation in PVN of stress responses, emotional regulation, and its indirect influence on the brain's reward system through neurotransmitter pathways, particularly DA, highlight its multifaceted role in addiction. The interplay between different types of neurons within the PVN, such as CRF, OXT, and AVP neurons, and their respective contributions to stress response, social behavior, and addiction, provide valuable insights into the neurobiological mechanisms of addiction. This understanding opens avenues for potential therapeutic interventions targeting the PVN and its neuronal pathways. The findings suggest that modulating the activity of PVN neurons could offer new strategies for treating drug addiction, emphasizing the need for further research in this area to develop more effective treatment modalities.

## AUTHOR CONTRIBUTIONS

Conceptualization, Yixiao Luo and Chang Yang; writing—review and editing, Chang Yang, Yixiao Luo, Dan Tao, Cuijie Shi, and Shihao Huang; supervision, Chang Yang and Yixiao Luo. All authors have read and agreed to the published version of the manuscript.

## CONFLICT OF INTEREST STATEMENT

The authors declare no conflicts of interest.

## Data Availability

All data generated or analyzed in this study are available from the corresponding author upon reasonable request.
